# Plasma Concentrations of sTREM-1 as Markers for Systemic Adverse Reactions in Subjects Treated With Weekly Rifapentine and Isoniazid for Latent Tuberculosis Infection

**DOI:** 10.3389/fmicb.2022.821066

**Published:** 2022-03-03

**Authors:** Tsai-Yu Wang, Jia-Yih Feng, Chin-Chung Shu, Susan Shin-jung Lee, Chung-Yu Chen, Yu-Feng Wei, Chih-Bin Lin, Wei-Chang Huang, Wei-Juin Su, Shu-Min Lin

**Affiliations:** ^1^Department of Thoracic Medicine, Chang Gung Memorial Hospital Linkou Main Branch, Taoyuan, Taiwan; ^2^School of Medicine, Chang Gung University, Taoyuan, Taiwan; ^3^Department of Chest Medicine, Taipei Veterans General Hospital, Taipei, Taiwan; ^4^Faculty of Medicine, School of Medicine, National Yang-Ming Chiao-Tung University, Taipei, Taiwan; ^5^Department of Internal Medicine, National Taiwan University Hospital, Taipei, Taiwan; ^6^Division of Infectious Diseases, Kaohsiung Veterans General Hospital, Kaohsiung, Taiwan; ^7^Division of Pulmonary and Critical Care Medicine, Department of Internal Medicine, National Taiwan University Hospital Yunlin Branch, Yunlin, Taiwan; ^8^Division of Chest Medicine, Department of Internal Medicine, E-Da Hospital, I-Shou University, Kaohsiung, Taiwan; ^9^Division of Chest Medicine, Department of Internal Medicine, Hualien Tzu Chi Hospital, Hualien, Taiwan; ^10^Division of Chest Medicine, Department of Internal Medicine, Taichung Veterans General Hospital, Taichung, Taiwan; ^11^Department of Post-Baccalaureate Medicine, College of Medicine, National Chung Hsing University, Taichung, Taiwan; ^12^Ph.D. Program in Translational Medicine, National Chung Hsing University, Taichung, Taiwan; ^13^School of Medicine, Chung Shan Medical University, Taichung, Taiwan; ^14^Department of Medical Technology, Jen-Teh Junior College of Medicine, Nursing and Management, Miaoli, Taiwan; ^15^Master Program for Health Administration, Department of Industrial Engineering and Enterprise Information, Tunghai University, Taichung, Taiwan

**Keywords:** exacerbation, sTREM-1, sTREM-2, LTBI, tuberculosis

## Abstract

**Background:**

A regimen of once-weekly rifapentine plus isoniazid for 3 months (3HP) is an effective treatment for subjects with latent tuberculosis infection; however, no reliable biomarker exists for predicting systemic adverse reactions (SARs) to 3HP treatment.

**Methods:**

This prospective, multi-center study evaluated the plasma concentrations of soluble triggering receptors expressed on myeloid cells (sTREM)-1 and sTREM-2 in subjects undergoing 3HP treatment and examined the associations between these biomarkers and SARs.

**Results:**

This study enrolled 80 consecutive subjects receiving 3HP treatment, 25 of whom had SARs and 55 of whom did not. Subjects with SARs presented higher concentrations of sTREM-1 at baseline than those without SARs (240.1 ± 19.1 vs. 176.7 ± 9.4 pg/mL, *P* = 0.001). The area under the receiver operating characteristic curves revealed that day 1 plasma levels of sTREM-1 (0.708, 95% CI, 0.584–0.833, *P* = 0.003) and sTREM-2 (0.343, 95% CI, 0.227–0.459, *P* = 0.025) as well as the sTREM-1/sTREM-2 ratio (0.748, 95% CI, 0.638–0.858, *P* = 0.001) had modest discriminative power pertaining to the development of SARs. An sTREM-1 level exceeding the cut-off value (>187.4 pg/mL) (hazard ratio [HR], 6.15; 95% CI 1.67–22.70, *P* = 0.006) and a sTREM-2 below the cut-off value (<237.2 pg/mL) (HR, 4.46; 95% CI 1.41–14.1, *P* = 0.011) were independent predictors of SARs after controlling for other variables.

**Conclusions:**

Plasma sTREM-1 and sTREM-2 levels are useful biomarkers for predicting SARs during 3HP treatment.

**Clinical trial government:**

NCT04655794

## Introduction

Tuberculosis (TB) is the leading cause of infectious disease-associated death worldwide, with 9 million new cases and nearly 2 million deaths reported annually (Dye et al., 1999). Treating high-risk patients for latent *M. tuberculosis* infection (LTBI) before the disease progresses to the active stage is a crucial strategy for controlling and eliminating TB (Rose, 2000; Cohn et al., 2020). A 9-month regimen of daily isoniazid (9H) has long been used as a treatment; however, it is associated with liver toxicity and high treatment interruption rates (Hirsch-Moverman et al., 2008). A regimen of once-weekly rifapentine plus isoniazid for 3 months (3HP) is an effective treatment for patients with LTBI, as the efficacy is similar to that of the 9H regimen but with a shorter treatment duration as well as higher adherence and treatment completion (Sterling et al., 2011). Nonetheless, recent studies have reported that the 3HP regimen is associated with systemic drug reactions (SDRs), such as flu-like syndrome (Sterling et al., 2015). One study on patients undergoing 3HP treatment reported that 8.2% experienced drug-related adverse events and 4.9% discontinued treatment due to adverse events (Sterling et al., 2011). Due to the shorter treatment duration and higher completion rate, there is a trend in increasing use of 3HP regimen for treatment of LTBI in medical practice. However, the occurrence of adverse reactions has been reported to be the major cause of treatment discontinuation in patients on the 3HP regimen (Sterling et al., 2011).

The mechanisms underlying the development of SDRs have yet to be clearly defined. Recent studies on 3HP as a treatment for LTBI have reported inconsistencies in symptoms upon single and multiple drug rechallenge. Note that many patients completed treatment despite adverse drug reactions (Schechter et al., 2006). This suggests that SDRs and flu-like symptoms are not necessarily immunologically mediated. One study reported that a 3HP regimen in conjunction with antiretroviral agents was correlated with a high incidence of flu-like symptoms and the acute release of inflammatory cytokines, including tumor necrosis factor-α (TNF-α), interferon-γ, and C-reactive protein (CRP) (Brooks et al., 2018). Thus, it appears that the development of SDRs in patients undergoing 3HP treatment may be attributed to inflammatory responses.

Triggering receptors expressed on myeloid cells-1 (TREM-1) is constitutively expressed on human monocytes/macrophages and neutrophils and is up-regulated by stimuli with lipopolysaccharide (LPS) binding to the TLR4. TREM-1 amplify inflammation by increasing the production of inflammatory cytokines (e.g., TNF-α, IL-1β, and IL-6) synergistically with toll-like receptor (TLR) and NOD-like receptor (NLR) signaling (Ornatowska et al., 2007; Dower et al., 2008; Thankam et al., 2016; Cao et al., 2017). Recent studies have also suggested that plasma sTREM-1 levels could potentially be used as a biomarker for disease severity and treatment outcomes in patients with pulmonary TB and latent TB (Shu et al., 2016; Feng et al., 2018). Signaling mediated by TREM-2 and DAP12 promotes phagocytosis and dampens TLR signaling, which reduces the production of proinflammatory cytokines (Thankam et al., 2016). By blocking systemic inflammation, the effects of TREM-2 activation are essentially the opposite of the effects of TREM-1 activation. The TREM-1/TREM-2 ratio has been identified as a reliable indicator of inflammation intensity associated with many diseases (Nguyen et al., 2015; Suchankova et al., 2020). However, researchers have yet to determine whether plasma sTREM-1, sTREM-2, or TNF-α can be used as biomarkers to predict the occurrence of systemic adverse reactions (SARs) during 3HP treatment.

This prospective multicenter observational study measured plasma concentrations of sTREM-1, sTREM-2, sTLR4, and TNF-α in patients undergoing 3HP treatment regimens and examined the independent association between these biomarkers and SARs. These biomarkers were also serially measured to determine their correlation with changes in SARs throughout the course of 3HP treatment.

## Materials and Methods

This prospective multicenter observational study was performed using data from eight medical centers in Taiwan covering the period from January 2017 to August 2019. The local Ethics Committee of Chang Gung Memorial Hospital approved the research protocol (NCT04655794), and each patient provided written informed consent.

### Study Population

Subjects newly identified with LTBI who elected to receive LTBI preventive therapy *via* 3HP were eligible for enrolment. Indications for LTBI screening included close contact with patients with active TB, patients with autoimmune diseases preceding biological therapy, healthcare workers, and subjects with other clinical conditions that increased the risk of LTBI. Diagnosis of LTBI was confirmed using the QuantiFERON-TB Gold In-Tube test (QFT-GIT; Qiagen, Valencia, CA, United States) with a cut-off value of 0.35 IU/mL. Exclusion criteria included age younger than 20 years, pregnancy, obesity, active TB or suspected of active TB during the clinical evaluation, close contact with a patient with multidrug-resistant TB, and previous instances of severe liver disease, end-stage renal disease (ESRD), or organ transplantation.

### Definition

Systemic adverse reactions were defined as adverse reactions of grade 2 or higher (Feng et al., 2020). Grade 2 SARs are defined as moderate, which indicates minimal, local, or non-invasive interventions as well as limiting age-appropriate instrumental activities of daily living (ADL). Grade 3 SARs are defined as severe or medically significant but not immediately life-threatening, which indicates hospitalization or prolongation of hospitalization, disabling, and limiting self-care ADL. Grade 4 SARs are life-threatening consequences in which urgent intervention is indicated. Grade 5 SARs are death related to adverse events. The investigator in charge determined whether SARs developed in relation to the LTBI treatment regimen. Patients visited outpatient clinics at weeks 2, 4, 8, and 12 for clinical evaluations and blood tests. The occurrence of adverse reactions was recorded during every visit throughout the treatment period. SARs severity was graded in accordance with the Cancer Therapy Evaluation Program Common Toxicity Criteria, version 5.0 (Cancer Therapy Evaluation Program, 2020). Hepatotoxicity was defined as an alanine aminotransferase (ALT) level greater than 3 times the upper limit of normal (ULN) with symptoms of nausea, vomiting, fatigue, or jaundice, or ALT levels greater than 5 times the ULN (Bliven-Sizemore et al., 2015).

### Study Design

This study assessed whether baseline plasma soluble TREM-1 (R&D, United States), soluble TREM-2 (RayBiotech, United States), soluble TLR4 (MyBioSource, United States), CRP (R&D, United States), and TNF-α (R&D, United States) could be used to predict the occurrence of SARs during 3HP treatment. In Taiwan, the directly observed treatment short-course (DOTS) program was started in 2006 (Lo et al., 2011) All LTBI patients were included in the DOTS program during the study period. All medical expenses related to the 3HP regimen and the DOTS program were paid for by the government. Blood samples obtained on day 1 and day 14 were used for plasma collection to identify instances of SARs side effects of grade 2 or higher. Soluble TREM-1, soluble TREM-2, soluble TLR4, CRP, and TNF-α plasma concentrations were obtained using ELISA in accordance with the manufacturer’s instructions. We compared the plasma levels of mediators between subjects with and without SARs (≥grade 2 side effects), between days 1 and 14 as well as at SARs.

### Statistical Analysis

Data are expressed as the standard error of the mean (SEM). The student’s *t*-test was used to compare continuous variables between the two groups, and the Mann-Whitney *U* test was used to deal with non-normal distributions. Categorical variables were compared using the chi-squared or Fisher’s exact test. Univariate analysis was used primarily for the selection of variables based on a *P* value of less than 0.1. Selected variables, including age, sTREM-1, and sTREM-2, were entered into a Cox proportional hazards model to identify the net effects of each individual factor. Hazard ratios (HRs) with 95% confidence intervals (CIs) were used to assess the independent contributions of significant factors. Receiver operating characteristic (ROC) curves were plotted to illustrate the predictive values of day 1 sTREM-1 and sTREM-2 on the development of SARs. We also calculated the respective areas under the curves. A *P* value of less than 0.05 was considered statistically significant. Analysis was conducted using SPSS (version 15.0; SPSS; Chicago, IL, United States).

## Results

### Clinical Characteristics and Adverse Effects

**FIGURE 1:**
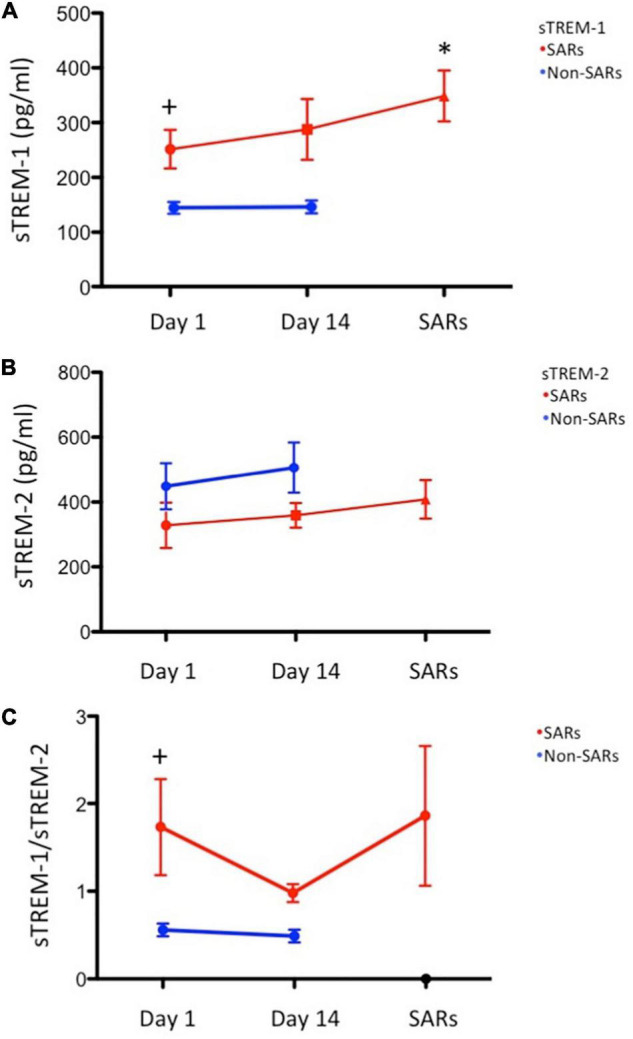
Serial sTREM plasma levels. (*N* = 12: systemic adverse reactions (SARs) group; *N* = 31: non-SARs group): **(A)** Mean sTREM-1 plasma levels were significantly higher in the SARs group (red circle) than in the non-SARs group (blue circle) on day 1 (*P* = 0.006). Mean sTREM-1 plasma levels were significantly higher on the day of SARs development (red circle) than that on day 1 (*P* = 0.003); **(B)** Mean sTREM-2 plasma levels in the SARs group (red circle) were not significantly higher than in the non-SARs group (blue circle) on day 1; **(C)** The sTREM-1/sTREM-2 ratio in the SARs group (red circle) was significantly higher than in the non-SARs group (blue circle) on day 1. *P < 0.05 versus day 1 of SAR group, ^+^*P* < 0.05 versus day 1 of Non-SAR group.

This study enrolled 80 consecutive subjects receiving 3HP treatment, 25 of whom had SARs and 55 of whom did not. The timing of SARs after the initiation of therapy was 17.2 ± 14.0 days. Table 1 lists the baseline demographics and clinical characteristics. The mean ages of subjects were as follows: with SARs (57.2 years) and without SARs (48.7 years). The distribution of male subjects was as follows: with SARs (41.7%) and without SARs (48.4%). The two groups were similar in terms of comorbidity frequency. sTREM-1 levels in the non-SARs group differed from those in the SARs group on day 1 (176.7 ± 9.4 vs. 240.1 ± 19.1 pg/mL, *P* < 0.001). The sTREM-1/sTREM-2 ratio was lower in the non-SARs group (0.7 ± 0.1 vs. 1.1 ± 0.1, *P* < 0.001) than in the SARs group; however, sTREM-2 levels were higher (484.9 ± 59.5 vs. 237.4 ± 12.6 pg/mL, *P* = 0.007). The two groups were similar in terms of sTLR4, TNF-α, and CRP levels. In subjects with SARs, the concentration of sTREM-1 on the day of SARs development (348.6 ± 46.5 pg/mL) was significantly higher than on day 1 (251.7 ± 35.3 pg/mL) and day 14 (287.8 ± 55.3 pg/mL vs. *P* = 0.006) (Figure 1). By contrast, the concentration of sTREM-1 in the non-SARs group did not differ between day 1 and day 14 (144.2 ± 12.4 pg/mL vs. 146.1 ± 12.5 pg/mL). The concentration of sTREM-2 in the SARs group was not significantly different among on the day of SARs development, day 1, and day 14 (408.6 ± 59.3 pg/mL vs. 328.5 ± 70.1 pg/mL vs. 359.2 ± 38.2 pg/mL, *P* = 0.198). The concentration of sTREM-2 in the non-SARs group did not differ significantly between day 1 and day 14 (450.5 ± 76.7 pg/mL vs. 512.3 ± 83.0 pg/mL) (Figure 1). The incidence of autoimmune disease was similar in patients with SARs vs. without SARs (Table 1). The baseline levels of sTREM-1, sTREM-2, sTREM-1/sTREM-2, sTLR4, TNF-α, and CRP were similar in patients with and without autoimmune disease (Supplementary Table 1).

**TABLE 1:** Demographic and clinical characteristics of patients.

Characteristics	Non-SARs *n* = 55	SARs *n* = 25	*p* value
Age (years)	48.7 ± 2.3	57.2 ± 3.6	0.023
Male	15 (48.4)	5 (41.7)	0.745
BMI (kg/m^2^)	24.6 ± 0.5	23.6 ± 0.6	0.836
Smoking	18 (32.7)	12 (48.0)	0.219
**Comorbidities**			
Asthma	1 (1.8)	0 (0)	0.999
HBV	3 (5.5)	1 (4.0)	0.999
HCV	2 (3.6)	0 (0)	0.999
HIV	1 (1.8)	0 (0)	0.999
Autoimmune disease	4 (7.2)	2 (8.0)	0.999
**Laboratory data baseline**			
AST, U/L	24.9 ± 2.2	26.0 ± 2.7	0.769
ALT, U/L	23.9 ± 2.9	25.8 ± 4.2	0.713
Total bilirubin, mg/dL	0.6 ± 0.1	0.7 ± 0.1	0.542
Creatinine, mg/dL	0.6 ± 0.1	1.2 ± 0.4	0.899
sTREM-1, pg/ml	176.7 ± 9.4	240.1 ± 19.1	0.001
sTREM-2, pg/ml	484.9 ± 59.5	237.4 ± 12.6	0.007
sTREM-1/sTREM-2	0.7 ± 0.1	1.1 ± 0.1	0.001
sTLR4, ng/ml	1.7 ± 0.5	1.8 ± 0.9	0.917
TNF-α, pg/ml	3.5 ± 0.3	4.2 ± 0.3	0.137
CRP, mg/ml	3.7 ± 2.5	4.3 ± 5.2	0.907

*Data are presented as mean (SEM) or n (%). BMI, body mass index; HBV, hepatitis B virus; HCV, hepatitis C virus; HIV, human immunodeficiency virus; SARs, systemic adverse reactions ≥ grade 2; AST, Aspartate aminotransferase; ALT, Alanine aminotransferase.*

Table 2 lists data pertaining to adverse events. In the SARs group, the most common adverse events associated with the gastrointestinal system were nausea and vomiting (48%) and anorexia (36%). The most common flu-like symptoms were fatigue (68%) and fever (52%). The percentage of abnormal liver function was similar in both groups.

**TABLE 2:** List of adverse events.

Characteristic	Non-SAR *n* = 55	SAR *n* = 25	*p* value
Adverse event	Grade 1	≥Grade2	
**Gastrointestinal reaction**			
Abdominal pain	0 (0)	8 (32.0)	0.001
Nausea/vomiting	2 (3.6)	12 (48.0)	0.001
Anorexia	1 (1.8)	9 (36.0)	0.001
**Flu-like symptoms**			
Fatigue	3 (5.5)	17 (68.0)	0.001
Dizziness	4 (7.3)	12 (48.0)	0.001
Headache	1 (1.8)	8 (32.0)	0.001
Fever	2 (3.6)	13 (52.0)	0.001
Myalgia/arthralgia	1 (1.8)	9 (36.0)	0.001
**Hypersensitivity reaction**	1 (1.8)	1 (4.0)	0.037
**Elevated liver enzyme levels**			
Any	2 (3.6)	1 (4.0)	0.999
1-3 ULN	2 (3.6)	1 (4.0)	0.999
3-5 ULN	0 (0)	0 (0)	0.999
>5 ULN	0 (0)	0 (0)	0.999
**Other drug reactions**	0 (0)	1 (4.0)	0.168

*Data are presented as n (%); SARs, systemic adverse reactions ≥ grade 2.*

### Area Under the Receiver Operating Characteristic Curve for Predicting Systemic Adverse Reactions

The areas under the ROC curves (AUROCs) revealed that day-1 plasma levels of sTREM-1 (0.708, 95% CI, 0.584–0.833, *P* = 0.003) and sTREM-2 (0.343, 95% CI, 0.227–0.459, *P* = 0.025) as well as the sTREM-1/sTREM-2 ratio (0.748, 95% CI, 0.638–0.858, *P* = 0.001) had modest discriminative power pertaining to the development of SARs (Table 3). The cut-off values for sTREM-1, sTREM-2, and the sTREM1/sTREM-2 ratio in predicting SARs were 187.4 pg/mL, 237.2 pg/mL, and 0.698, respectively.

**TABLE 3:** Area under the receiver operating characteristic curves for serum levels of sTREM-1 and sTREM-2 on day 1 for the prediction of SARs.

	Area (95% CI)	*P*-value	Cut-off value	Sensitivity	Specificity
sTREM-1	0.708 (0.584–0.833)	0.003	187.4 pg/ml	0.760	0.618
sTREM-2	0.343 (0.227–0.459)	0.025	237.2 pg/ml	0.680	0.425
sTREM-1/sTREM-2	0.748 (0.638–0.858)	0.001	0.698	0.800	0.636

*SARs: systemic adverse reactions ≥ grade 2.*

### Cox Proportional Hazards Analysis for Predicting Systemic Adverse Reactions

Univariate analysis was used for the selection of variables based on a *P* value of less than 0.1. We entered the variables (age, sTREM-1 levels higher than the cut-off value of >187.4 ng/mL, and sTREM-2 levels less than the cut-off value of <237.2 ng/mL) into a Cox proportional hazard model (Table 4). After controlling for other variables, sTREM-1 levels (HR, 6.15; 95% CI 1.67–22.70, *P* = 0.006) and sTREM-2 levels (HR, 4.46; 95% CI 1.41–14.12, *P* = 0.011) remained significant predictors of SARs. Note that after adjusting for the effects of other factors, age was not significantly predictive of SARs occurrence.

**TABLE 4:** Cox proportional hazards analysis of risk factors for SARs.

	Univariate			Multivariate		
Factors	HR	95% CI	*P* value	HR	95% CI	*P* value
Age	0.96	0.93–0.99	0.027	0.97	0.93–1.01	0.126
sTREM-1 > cut-off value	6.30	1.91–20.79	0.003	6.15	1.67–22.70	0.006
sTREM-2 < cut-off value	2.96	1.09–8.01	0.033	4.46	1.41–14.12	0.011

*SARs, systemic adverse reactions ≥ grade 2.*

### Association Between Plasma sTREM-1 and Adverse Reactions

To elucidate the relationship between sTREM-1 and adverse reactions, we compared plasma sTREM-1 levels with several related variables (Table 5). Plasma sTREM-1 levels were correlated with nausea and vomiting (*r* = 0.269, *P* = 0.016) and fatigue (*r* = 0.278, *P* = 0.013) but not with abdominal pain, anorexia, dizziness, headache, myalgia, arthralgia, or fever.

**TABLE 5:** Linear regression analysis of day 1 sTREM-1 versus variables of adverse reactions.

Variables	Correlation (r)	*P* value
**Gastrointestinal reaction**		
Abdominal pain	0.206	0.067
Nausea/vomiting	0.269	0.016
Anorexia	0.088	0.439
**Flu-like symptoms**		
Fatigue	0.278	0.013
Dizziness	0.038	0.735
Headache	0.126	0.265
Myalgia/arthralgia	0.057	0.618
Fever	0.164	0.146

## Discussion

Systemic adverse reactions are common in patients receiving 3HP treatment for LTBI. Baseline sTREM-1 levels and the sTREM-1/sTREM-2 ratio were higher in subjects with SARs than those in subjects without SARs; however, sTREM-2 levels were lower. We also observed significantly higher sTREM-1 concentrations at exacerbation than at baseline. Taken together, sTREM-1 and sTREM-2 plasma levels were independent predictors of SARs development. ROC curves revealed that sTREM-1 and sTREM-2 levels as well as the sTREM-1/sTREM-2 ratio at day 1 had moderate discriminative power in predicting the development of SARs.

A regimen of weekly rifapentine plus isoniazid is as effective as a 9-month regimen of daily isoniazid in treating LTBI in high-risk subjects; however, 3HP treatment regimen commonly leads to the occurrence of SARs. Researchers have yet to elucidate the possible mechanisms underlying SARs development. One recent study examined potential mechanisms underlying SARs development in healthy volunteers undergoing a once-weekly regimen of isoniazid and rifapentine with an antiretroviral medicine, dolutegravir (Brooks et al., 2018). They determined that the SARs symptoms were associated with elevated cytokine plasma levels, including TNF-α, CRP, and IFN-γ. It appears that inflammatory responses may play an important role in mediating SARs in drug-induced adverse reactions. Further studies are required to identify the mechanism underlying the development of SARs in patients undergoing treatment using rifapentine plus isoniazid.

In the current study, patients who developed SARs during the 3HP regimen had higher baseline sTREM-1 and lower sTREM-2 levels than those without SARs. Day-1 sTREM-1 and sTREM-2 levels were independent predictors of SARs development. sTREM-1 levels at the time of exacerbation were significantly higher than those at baseline. TREM-1 amplifies inflammation by increasing inflammatory cytokine levels *via* TLR-4 and NLR signaling (Ornatowska et al., 2007; Dower et al., 2008; Thankam et al., 2016; Cao et al., 2017). This indicates that inflammation associated with TREM-1 may play a key role in the development of SARs in patients receiving 3HP treatment for LTBI. Elevated sTREM-1 levels have been identified in infectious and non-infectious inflammatory diseases (Cao et al., 2017). In fact, plasma sTREM-1 has been identified as a potential biomarker for disease severity and treatment outcomes in patients with pulmonary TB. sTREM-1 levels are associated with TB-related constitutional symptoms, such as poor appetite (Feng et al., 2018). Plasma sTREM-1 levels are also an independent risk factor for persistent positive readings in interferon-gamma release assays in cases of LTBI (Shu et al., 2016). Note, however, that this is the first study to identify sTREM-1 and sTREM-2 levels as independent predictors of SARs in patients with LTBI.

Our study showed that development of SARs was associated with increased plasma levels of sTREM-1 and sTREM-1/sTREM-2 ratio but not with plasma levels of CRP, sTLR-4, and TNF-α. The possible causes for the different results comparing with previous report may be attributed by distinct study populations. Our study recruited patients receiving 3HP treatment while their study recruited patients receiving combination of 3HP and antiretroviral treatment. In addition, TREM-1 activation was shown to result in a persistent release of cytokines and chemokines (TNF-α, IL-1β, IL-8, and monocyte chemotactic protein [MCP]-1), (Cao et al., 2017). Thus, further studies are required to identify the mechanism underlying TREM-1 associated development of SARs in patients undergoing treatment using rifapentine plus isoniazid.

Evidence revealed that sTREM-1 levels are associated with the disease activity of autoimmune disease (Bassyouni et al., 2017). Our results showed similar incidences of autoimmune disease in SARs and non-SARs groups. The baseline levels of sTREM-1, sTREM-2, and sTREM-1/sTREM-2 were similar in patients with and without autoimmune disease. Therefore, the different levels of sTREM-1, sTREM-2, and sTREM-1/sTREM-2 ratio in SARs vs. non-SARs groups are not affected by comorbidity of autoimmune disease.

We observed elevated plasma sTREM-1 and sTREM-2 levels and a higher sTREM-1/sTREM-2 ratio in patients with LTBI who experienced SARs after 3HP treatment. The corresponding AUROC identified plasma sTREM-1 and sTREM as modest predictors of SARs development after 3HP treatment. If further large-scale prospective studies provide corroborative evidence, these findings could have substantial clinical implications. Patients could have their sTREM-1 and sTREM-2 levels measured prior to undergoing 3HP therapy. In cases where the levels are high, the patient could be monitored more frequently and perhaps treated *via* 3HP to improve compliance and clinical outcomes. Nonetheless, further studies will be required to evaluate the efficacy of this approach.

Emerging evidence suggests that TREM-2 activation inhibits the production of proinflammatory cytokines and promotes phagocytosis in macrophages. This implies that TREM-2 activation has the opposite effect of TREM-1 activation; i.e., blocking the development of systemic inflammation (Ito and Hamerman, 2012). The elevated sTREM-1/sTREM-2 ratio in the blood indicates that enhanced systemic inflammation may play an essential role in the development of SARs during 3HP treatment.

sTREM-1 is released from myeloid cells in response to toll-like receptor-4 (TLR4) activation (Netea et al., 2006). In addition, TREM-1 has the synergistic ability to amplify the signaling of the TLR4, which can recognize components of a variety of microorganisms including bacteria, fungi, and viruses (Netea et al., 2006; Fortin et al., 2007). Therefore, TREM-1 expression is closely linked to TLR4 activity. However, our results showed 3HP treatment related SARs are correlated with sTREM-1 plasma level but not with sTLR4 levels. According to previous studies, these interaction of TREM-1 expression and activation are mediated through the binding of membrane-bound TLR4 molecule. Soluble TLR4 are considered structurally identical to their membrane bound counterparts but do not participate in the TLR pathway. Instead, they reduce inflammatory responses by competing with TLRs for ligands (Hyakushima et al., 2004). The study was designated to measure the potential plasma biomarkers for prediction of SARs in 3HP regimen, thus, we measure the plasm levels of sTLR rather than the expression of membrane-bound TLR4. This may explain the reason why the levels of sTREM-1 but not sTLR4 are predictors for SARs in 3HP treatment.

This study had several limitations that should be considered in the interpretation of our results. First, the sample size was somewhat small, and we recruited only a specific subgroup of subjects with LTBI undergoing 3HP treatment. Previous studies reported higher baseline plasma sTREM-1 levels in patients with liver cirrhosis and ESRD (Shu et al., 2016). Thus, to avoid bias related to elevated baseline sTREM-1 levels in these subgroups, we excluded patients with liver cirrhosis and ESRD. We have yet to determine whether these markers identified in this study provide similar predictive power for other subpopulations receiving 3HP treatment for LTBI. In addition, the single-arm observational study of 3HP treatment in LTBI patients did not measure the role of those inflammatory biomarkers in LTBI patients receiving other treatment regimens. A randomized control study comparing the plasma levels of inflammatory biomarkers in patients receiving 3HP vs. 9H treatment is needed to elucidate the role of these biomarkers in LTBI patients receiving different treatment regimens.

Taken together, plasma sTREM-1 and sTREM-2 appear to be useful biomarkers for predicting the development of SARs during 3HP treatment. Further prospective studies with larger samples will be required to confirm these results. Nonetheless, the results of this study provide a basis for further research into the role of inflammation in adverse reactions associated with 3HP.

## Data Availability Statement

The datasets presented in this article are not readily available because of patient confidentiality and participant privacy. Requests to access the datasets should be directed to TYW (wang5531@gmail.com).

## Ethics Statement

The studies involving human participants were reviewed and approved by the Ethics Committee of Chang Gung Memorial Hospital approved the research protocol (NCT04655794). The patients/participants provided their written informed consent to participate in this study.

## Author Contributions

TYW and SML are the guarantors taking responsibility for the content of the manuscript and including all data and analysis. TYW, JYF, and CCS contributed directly to the study design and were responsible for the gathering of data. SSJL, CYC, YFW, CBL, WCH, and WJS contributed directly to data management and statistical analysis. All authors were substantially involved in the study design and drafting of the manuscript for intellectual content, and all authors reviewed the final manuscript prior to submission.

## Conflict of Interest

The authors declare that the research was conducted in the absence of any commercial or financial relationships that could be construed as a potential conflict of interest.

## Publisher’s Note

All claims expressed in this article are solely those of the authors and do not necessarily represent those of their affiliated organizations, or those of the publisher, the editors and the reviewers. Any product that may be evaluated in this article, or claim that may be made by its manufacturer, is not guaranteed or endorsed by the publisher.
