# Supplementary material for: Plasma Concentrations of sTREM-1 as Markers for Systemic Adverse Reactions in Subjects Treated With Weekly Rifapentine and Isoniazid for Latent Tuberculosis Infection
Source: Front Microbiol. 2022 Mar 3;13:821066. doi: 10.3389/fmicb.2022.821066 (PMC8927064; doi:10.3389/fmicb.2022.821066)
Supplement: Supplementary file 1 [file Table_1.pdf]

Supplement table 1. The plasma levels of biomarkers in patients with and without autoimmune disease

	Subjects with autoimmune disease n=6	Subjects without autoimmune disease n=74	p value
sTREM-1, pg/ml	223.3±38.9	194.3±9.6	0.412
sTREM-2, pg/ml	208.7±28.2	423.7±45.9	0.189
sTREM-1/sTREM-2	1.2±0.2	0.8±0.1	0.266
sTLR4, ng/ml	1.8±0.2	1.6±0.4	0.888
TNF-α, pg/ml	4.3±0.5	3.7±1.3	0.896
CRP, mg/ml	3.0±0.5	3.9±0.9	0.778

Data are presented as means ±SEM
